# Collaborative measurement of data opening policy in China’s municipal government data management system: Taking a regional central city as an example

**DOI:** 10.1371/journal.pone.0289550

**Published:** 2023-08-03

**Authors:** Yan Li, Qian Yang, Chunzi Wang, Yinhe Sun

**Affiliations:** School of Management, Xi’an Polytechnic University, Xi’an, China; Hebei Agricultural University, CHINA

## Abstract

In the current era of big data, the exponential increase in the volume of social data has exerted a significant influence on the government and all sectors of society, with the opening of government data becoming an irresistible trend. In this paper, several regional central cities are selected as the representatives of municipal governments to analyze the characteristics and weaknesses of their data-opening policies from various perspectives including policy effectiveness, policy tools, and policy targets. This is of reference significance for the municipal governments of an individual country in the formulation of data opening policies. To be specific, with 2015–2021 as the timeline, based on the six regions of the country, the central cities of each region are selected as representatives of municipal cities, and the text coding of 85 data opening policies implemented in regional central cities are performed. On this basis, a “policy tool-policy targets-policy effectiveness” collaborative measurement model is constructed through the policy tool theory, and the co-evolution analysis of the relevant policies is conducted. The research results are as follows. Firstly, there is a positive correlation between the total policy effectiveness and its quantity, indicating that the number of data-opening policies plays a positive role to a certain extent. However, the average effectiveness shows no significant increase, indicating the inadequate specificity of each policy. Secondly, the degree of synergy between policy tools fluctuates periodically, indicating that the government is constantly trying new methods, with more importance attached to the synergy between government capacity cultivation and positive incentive tools. Thirdly, policy targets continue to show new connotations over time, and there has been new progress made in the coordination between the other three objectives driven by data opening. However, it is imperative to enhance the synergy between the objectives of building a smart city and improving the services related to livelihood. Finally, some targeted suggestions are put forward on how to further improve the data opening policy implemented by municipal governments from three perspectives.

## 1 Introduction

With the improvement of computer storage capacity and the development of complex algorithms, the scale of data has increased exponentially, which drives a shift from the information age to the era of big data. Meanwhile, there is an inevitable trend of data openness. In the era of big data, the volume of data collected from all sectors of society and the government is increasing dramatically, with the government responsible for regulating data at all levels of society [1]. As data volume increases exponentially, the diversification of data continues, which compels the government to carry out effective data-open governance. With government data increasing continuously, there is a key issue facing the government about how to improve the transparency and openness of the government in making full use of data to serve the public.

The Chinese governments at all levels have successively published a series of relevant policies for effective governance over data openness. At the national level, the “Action Plan for Promoting Big Data Development” [2], “Outline of National Informatization Development Strategy” [3], and “Pilot Program for the Opening of Public Information Resources” [4] and other documents have been promulgated from 2015, specifying the need to improve the openness of government data. National policy support is effective in strengthening data openness and guiding both the public and enterprises to access and use government data. Concerning local governments, there have been positive responses made to the spirit reflected in national policy documents as both provincial and municipal cities have successively established a series of data-opening platforms, with substantial progress and good repercussions made in some places. However, there are still problems, such as the variation in levels of openness between some local governments [5], which is a critical constraint on the process of improving the data openness of local governments.

In this context, this paper aims to explore the current situation of municipal government data opening policy coordination across the country and provide reasonable suggestions for effective governance on this basis. From the perspective of policy synergy, the data opening policies enforced in six regional central cities of the country, namely, Shenyang, Nanjing, Wuhan, Shenzhen, Chengdu, and Xi’an, are taken as the research object in this paper to construct a “policy tool-policy goal-policy effectiveness” collaborative measurement model, for analyzing the evolution of municipal government policy coordination. On the one hand, a framework of three-dimensional analysis of municipal government data opening policies is constructed to quantify these policies in line with the quantitative standards formulated. On the other hand, a timeline is taken to analyze the co-evolution process of policy effectiveness, policy tools, and policy objectives.

First, we further improve the research object of the open government data policy research and study the policies of representative cities at the prefectural and municipal levels in China. Second, it enriches the analytical perspective of municipal government data openness policies and improves the analysis of the core contents of policy texts. Finally, it complements the synergistic effectiveness of municipal government data openness policies and deeply analyzes the internal synergy of representative municipal government data openness policies.

## 2 Literature review

### 2.1 Open data policy text analysis

Government data opening policy refers to a norm and guidance system developed by government departments for information disclosure, including various strategic plans, regulations, action guidelines, rules, procedures, etc. [6]. The representative existing research results about the analysis of data opening policies are listed in Table 1.

**Table 1:** Representative research results of government data open policy.

Type of study	Main research content	Representative scholars
Learning and comparison	Through an analysis of the opening-up policies adopted in developed countries such as the United States, France, the United Kingdom, Australia, Canada, etc., enlightenment and suggestions are provided for China.	Cai and Huang [7], Xiao et al. [8], Huang and Liu [9], Chen [10], Hu and Lin [11], etc.
Policy content analysis	Through textual dissection, the content or semantic implication of the policy is analyzed, such as the three perspectives of “policy type, organization, policy objectives”, “content analysis method,” “semantic network analysis,” and so on.	Tan and Liu [12], Chen and Zhao [13], Zhang [14], Jung and Park [15], etc.
Dimensional analysis	From two-dimensional analysis to three-dimensional analysis, it is diversified and improved. Such as the frameworks of two-dimensional analysis including “Policy Tools-Government Data Open and Shared Management,” “Instrumentality-Content Evaluation,” and the frameworks of three-dimensional analysis like “Policy Tools-Policy Forms-Policy Content,” “Policy Tools-Policy Objectives-Policy Strength.”	Meng and Wang [16], Tang et al. [17]; Zhao [18], Peng and Liu [19], etc.
Model quantitative evaluation	Quantitative policies are evaluated by constructing an evaluation framework or using an index model. The examples include “the evaluation framework composed of quality dimension and a series of compliance indicators,” “PMC index model,” “event history analysis method,” etc.	Viscusi et al. [20], Song et al. [21], Wang and Xiang [22], etc.

As shown in Table 1, scholars have gradually enriched their research on the text of data opening policies, showing a stepwise upward trend from directional analysis to framework analysis and then to quantitative analysis. At present, scholars mainly study four major aspects, some scholars analyze and compare the open policy with developed countries to draw inspiration and suggestions for China; some scholars conduct a deeper analysis of the policy content; some scholars gradually establish a two-dimensional framework and three-dimensional framework for the analysis of the policy content; some scholars also evaluate the policy quantitatively and use index models, such as the PMC model.Despite some significant progress made in four aspects, there is still no unified standard available for the analysis and research of government data opening policy. Therefore, a further discussion will be conducted in this article about the analysis and research of the policies on municipal government data openness.

### 2.2 Open data policy synergy analysis

As for the collaborative analysis of government data opening policies, scholars have carried out a lot of research both at home and abroad. In general, it mainly involves the calculation of the degree of collaboration, the construction of theories and frameworks, and the positioning of policy co-evolution objects. Table 2 lists the representative existing research results about the collaborative analysis of government data open policy.

**Table 2:** Representative research results of government data open policy coordination.

Type of Study	Main research content	Representative scholars
Basic concepts and connotations	The definition of basic concepts and relevant theories to policy coordination are elaborated, such as “concept definition,” “related theory,” “connotation analysis,” and so on.	Ma and Hong [23], Meijers and Stead [24], Camarero and Tamarit [25], etc.
Policy Synergy Calculation	Collaborative research on data openness policy and security policy or privacy protection policy, respectively, such as the synergy research on “openness and privacy protection,” “openness and security,” and other topics.	Zhou et al. [26], Zhang and Ma [27], etc.
Theoretical Perspective and Framework Construction	From the perspective of policy tool theory/policy synergy, a relevant framework is constructed and analyzed using a synergistic quantitative model. For example, the policy “effectiveness-tool” and “tool-target-strength” frameworks and model measures are applied to calculate policy synergy.	Zhang et al. [28], Hong and Ma [29], etc.
Policy co-evolution object positioning	Through dimension construction and analysis, the collaborative measurement model is constructed for the analysis of data openness collaborative policy, the research object is selected for analysis, and its importance is clarified, such as “Stake-related Theme Collaboration,” “Provincial and Municipal Level,” “Construction of Collaborative Mechanism,” etc.	Chen [30], Mao et al. [31], Bai et al. [32], etc.

By determining the concept and connotation of policy synergy, it can be seen from Table 2 that the research on synergy and quantification has gradually shown an upward trend, with policy research showing a stepwise rise from directional analysis to framework analysis and then to quantitative analysis. As the trend continues, scholars have gradually constructed the relevant measurement models and evaluation index systems for quantitative research on government data opening policies. Specifically, in terms of basic concept and connotation research, scholars mainly analyze the definition of concepts and related theories and connotation analysis; in terms of policy synergy calculation, they compare two categories of policy synergy; in terms of theoretical perspective and framework construction, they mainly use a combination of framework and quantitative model based on a certain theory/perspective to calculate policy synergy, this is because the collaborative quantitative measure can be used to describe the collaboration of data-opening policies more objectively and effectively, collaborative policies have more advantages than single policies, and practical policy collaboration is better at improving policy performance [33]; However, regarding the orientation of policy synergy evolution objects, it can be found that the objects of government data opening policy synergy research need to be further improved.

### 2.3 Research review

After the above summary, it can be found that there is still room for expansion of the current research on this aspect of China’s government data openness policy: First, it is found that the research objects of government data open policy research need to be further improved, and few studies have been conducted involving the policy research of sub-provincial cities or municipal cities in China. Secondly, the existing research has a single perspective on the analysis of municipal government data openness policy, and the core content of the policy text needs to be improved. Third, the existing research lacks attention to the synergy of municipal government data opening policies, and the research on internal synergy needs to be improved.

By sorting out domestic and foreign literature, it can be found that there is a need to further improve the objects of government data open policy collaborative research. Since few studies have paid attention to the research on collaborative policy measurement of sub-provincial or prefecture-level cities, this article focuses on the representative regional central cities of the municipal government to analyze the degree of coordination in the data opening policy adopted by municipal governments, which provides a valuable reference for improving the data openness of municipal governments.

## 3 Municipal open government data policy framework construction

**Fig 1:**
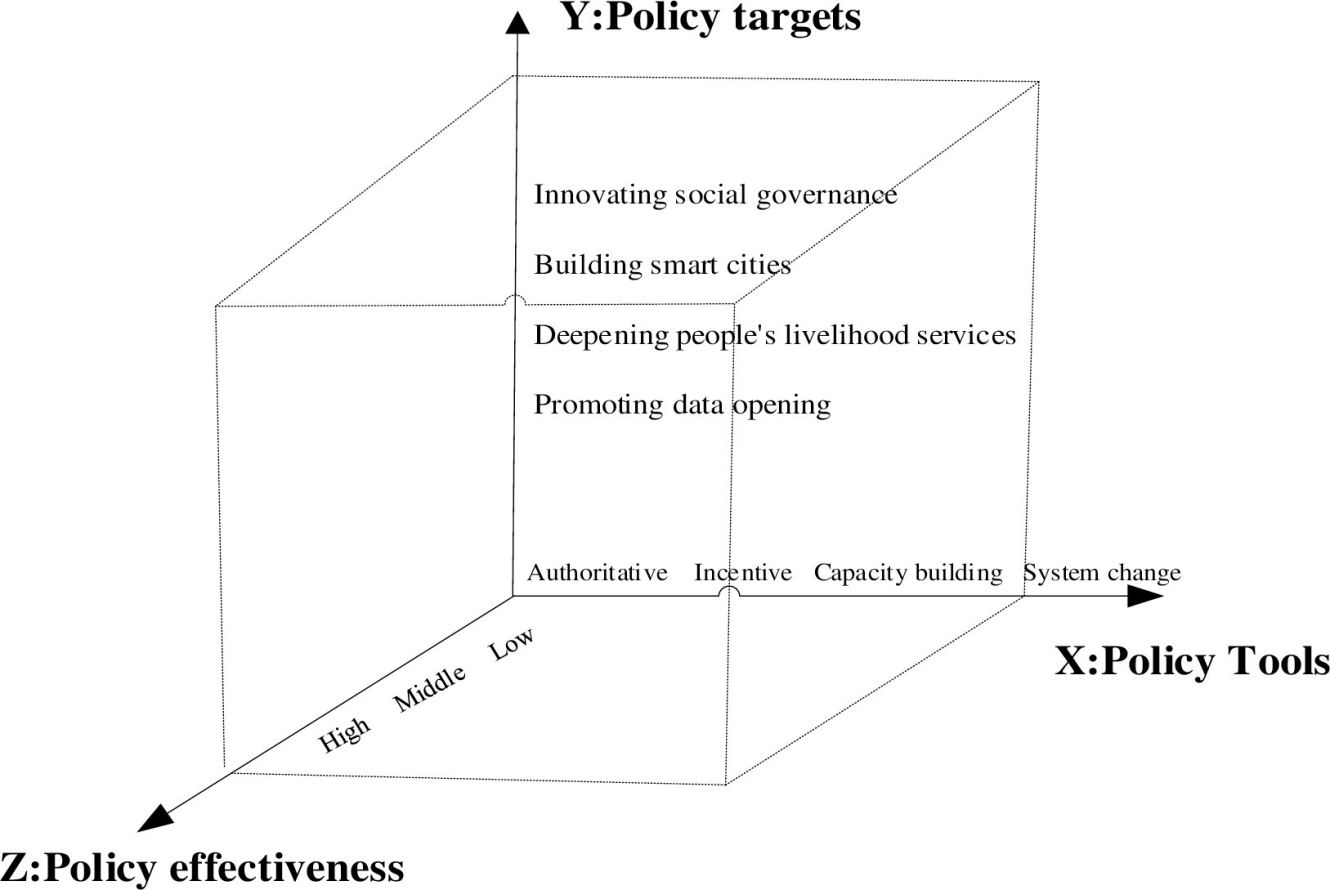
The three-dimensional framework of government data openness policy.

Peng et al. [34], Zhong et al. [35], and other scholars have conducted a study on the innovation policy in China from three perspectives: policy measures, policy objectives, and policy intensity. In this paper, the overall research of government data opening is carried out on three levels: policy tools, objectives, and effectiveness. Policy tools represent the measures taken to achieve policy objectives, and policy effectiveness is a measure used to represent the release level of policy objectives. On this basis, a three-dimensional framework of data openness policy analysis of “policy tools-policy objectives-policy effectiveness” is constructed (see Fig 1).

### 3.1 X-dimension: Policy tools

At present, many scholars have conducted research on the classification of policy tools. The following table summarizes the policy tools currently explored by some scholars based on the literature review (see Table 3).

**Table 3:** Introduction summary of classification and definition of policy tools.

Number	Scholar	Policy classification
1	Rothwell and Zegveld [36]	Supply Tools, Environmental Tools, and Demand Tools
2	McDonnell and Elmore [37]	Command tools, incentive tools, capacity-building tools, and system change tools
3	Schneide and Ingram [38]	Authoritative tools, ability tools, inducement tools, symbolic and persuasive tools, and learning tools
4	Howlett and Ramesh [39]	Mandatory, Hybrid, and Voluntary Instruments

Previously, most of the research on government data openness policy tools focused on the supply-type, environment-type, and demand-type tools, with little attention paid to the combination of multiple tools. To a certain extent, the comprehensive application of policy tools is ignored. Therefore, based on the classification theory of policy tools proposed by Schneider and Ingram, McDonnell, and Elmore, this study focuses on the open government data policy. The policy tools concerned in this study are divided into four categories: authority, incentives, capacity building, and system change. Moreover, the research of Huang et al. [40], Yao and Song [41], Liu [42], Wang et al. [43], and other scholars on policy analysis is drawn on to subdivide the policy tools studied in this paper for the multi-faceted and multi-level classification of policy instruments.

### 3.2 Y-dimension: Policy targets

With the State Council’s issuance of documents such as the Notice on the Issuance of Action Plan for Promoting the Development of Big Data, data openness has become one of the priorities of the government m. Currently, there has also been some significant progress made in the analysis and definition of policy targets, including the typical results of scholarly research (see Table 4).

**Table 4:** Quantitative criteria of policy targets.

Number	Scholar	Classification of policy targets
1	Peng and Liu [19]	Government data open sharing, big data applications, smart government architecture
2	Hong and Ma [29]	Optimize government governance, promote economic development, and innovate livelihood services
3	Tan and Liu [12]	Promote the development of big data, develop e-government, build smart cities, and improve government governance
4	Wang et al. [43]	Platform construction, livelihood services, economic integration, government applications

**Fig 2:**
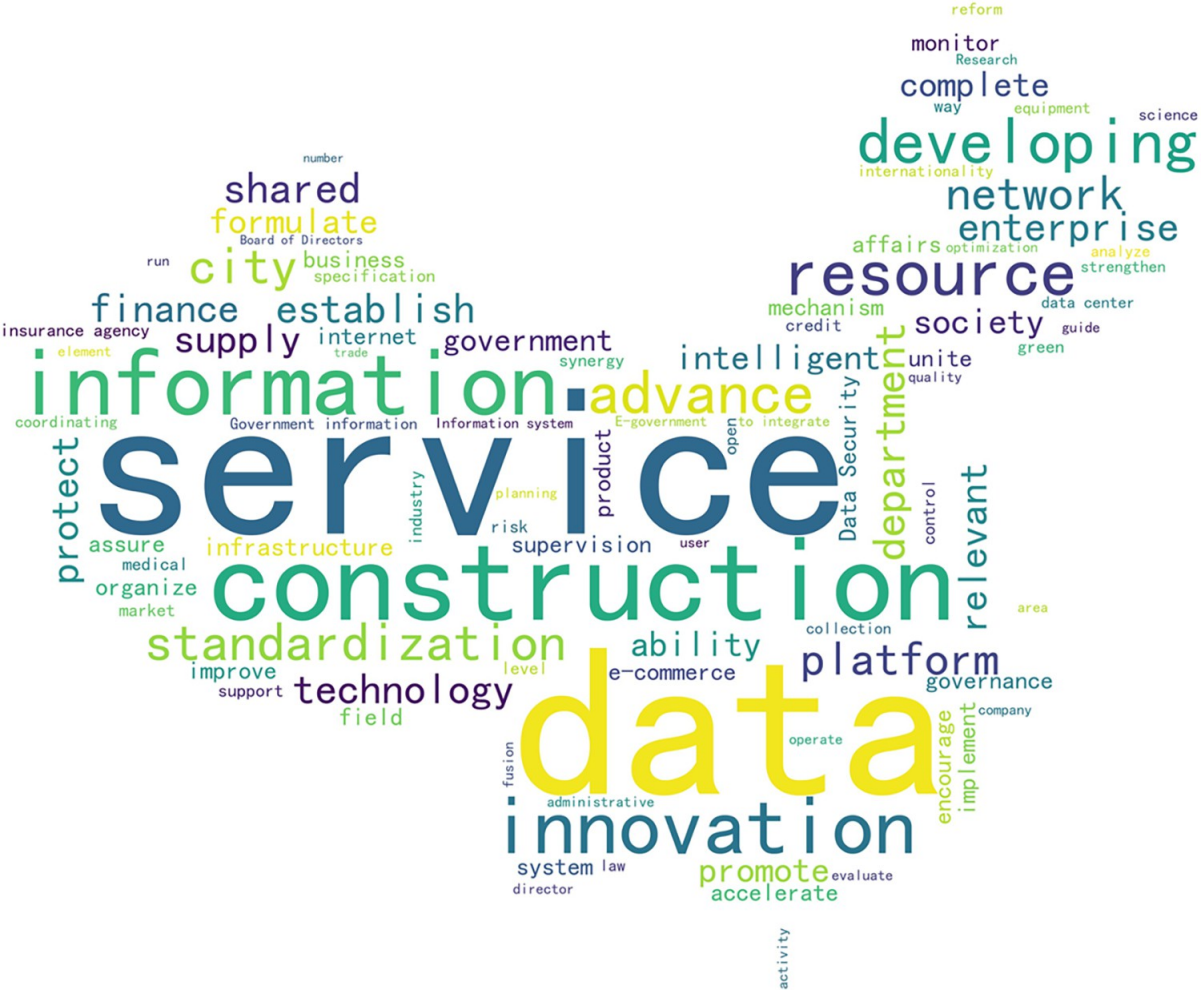
Regional central city open data policy word cloud.

Based on the above scholars’ definition of the goal of policy data opening, and the word cloud map of the regional central city policy texts collected in this paper (see Fig 2), the high-frequency words like “data,” “service,” “construction,” “innovation” and other are presented. After careful consideration given to these frequent words, the goals set by the municipal government for data opening policy are categorized into four classes: enhancing data openness, deepening livelihood-related services, building smart cities, and innovating social governance.

### 3.3 Z-dimension: Policy effectiveness

Taking into account the relevant provisions set out in the Regulations of “the State Council on Regulation Making Procedures” in 2018, the study of Yang et al. [44] on talent policy, and the types of policies collected from regional central cities and policy promulgating institutions, the final level classification applicable to the effectiveness of policies promulgated by municipal governments are divided into five tiers as follows from strong to weak (see Table 5).

**Table 5:** Efficacy dimension classification table.

Number	Level classification	Potency strength
1	Local regulations promulgated by the National People’s Congress and its Standing Committee	high
2	Regulations promulgated by the government	middle
3	Temporary regulations, plans, decisions, opinions, measures, detailed rules, plans, and standards promulgated by the government	middle
4	Opinions of committees, bureaus, and offices under the jurisdiction of the government	middle
5	Notices, announcements, etc.	low

## 4 Process and methodology

### 4.1 Research process

**Fig 3:**
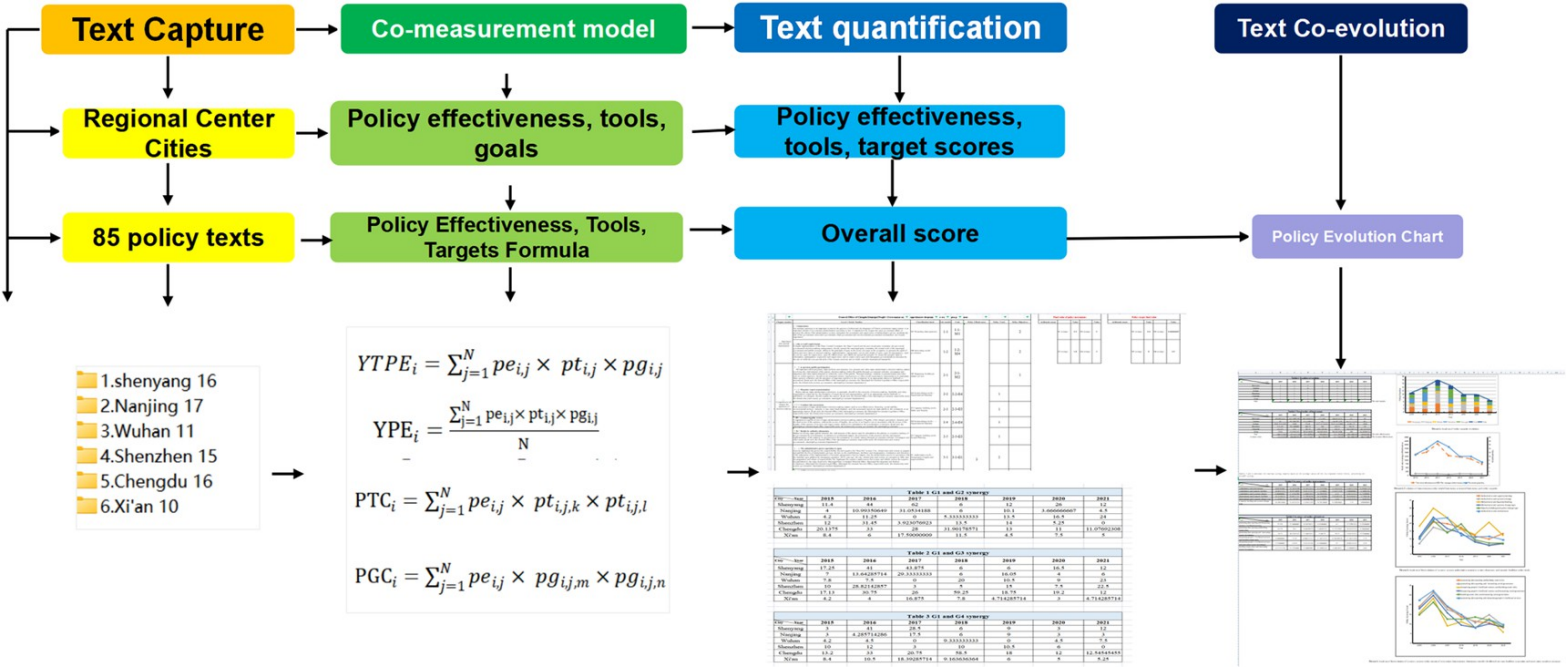
Schematic diagram of the research process.

The research process in this paper is divided into four main steps. First, the collection of texts is conducted. Second, the construction of the policy synergy model is conducted. Then, we develop quantitative criteria to code and score the policy texts, and coordinate with experts to adjust the scoring results. Finally, measurement quantification of text synergy effects is performed (see Fig 3).

**Fig 4:**
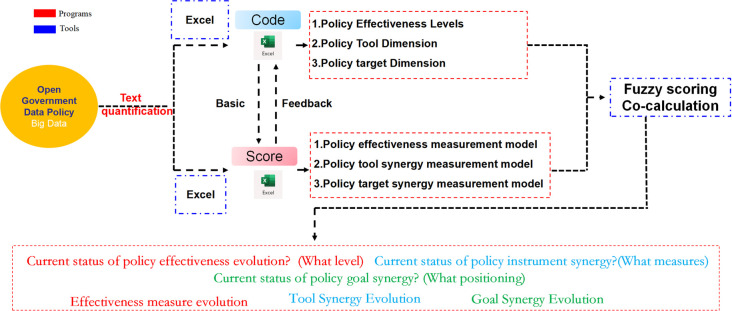
Schematic diagram of the calculation process.

In this paper, we use the measurement model to perform calculations and obtain the analysis results. The calculation process mainly includes text quantification and synergetic measurement calculation (see Fig 4). The text quantification calculation includes scoring the policy effects, policy instruments and policy objectives of each text separately, and then substituting into the synergy measurement formula for calculation.

### 4.2 The data

#### 4.2.1 Study subject selection

In this paper, the regional central cities specified in the “National Urban System Plan (2010–2020) (Draft)” are chosen to represent municipal governments. With the data openness policy adopted by the regional central city as the research object, the policy samples are mainly searched and collected from 2 databases: Beida Faber and the official website of the municipal government.

#### 4.2.2 Research data collection

(1) Preliminary collection

To achieve the research objectives set out in this paper, it is necessary to search and collect the relevant policies for the six central cities first. The process of doing this is as follows. Firstly, the keywords including “data opening,” “data sharing,” and “government data” are searched in the database of Beihang University. Secondly, the above keywords are entered again on the websites of municipal governments for a second-time search. In this paper, the time of “the State Council promulgating the Action Outline for Promoting the Development of Big Data” in 2015 is taken as the starting point to search, with the title and content as the two paths to finding the documents related to the corresponding six central cities from the two major databases and the official websites of each municipal government (between 2015 and October 27, 2021).

(2) Secondary screening

To improve the accuracy of source policy texts, three major screening criteria are adopted as the secondary screening guidelines after the initial search to obtain the texts, as detailed below.

1. Whether it is a policy in force such as notice, opinion, plan, etc.
2. Whether there are detailed policy requirements for the open data system.
3. Whether it is a specific city-level policy text.

There are 85 valid policy texts were finally included by following the above three criteria and excluding the policy texts that are not directly related to the theme of “government data openness,” such as meeting notices, approval letters, project declaration notices, and other inapplicable policy texts (see Table 6).

**Table 6:** Summary of open data government policies of regional central cities.

Country Region	Regional center cities	Number
Northeast Region	Shenyang (Liaoning Province)	16
East China	Nanjing (Jiangsu Province)	17
Central China	Wuhan (Hubei Province)	11
South China	Shenzhen (Guangdong Province)	15
Southwest Region	Chengdu (Sichuan Province)	16
Northwest China	Xi’an (Shaanxi Province)	10

### 4.3 Quantitative standard

#### 4.3.1 Quantitative criteriafor policy tools

In this paper, the ideas of Peng et al. [34] and Zhang et al. [45] are adopted to quantify the criteria of policy measures. Meanwhile, the quantitative criteria of policy instruments required for analysis in this paper are identified according to the level of detail and implementation of the policy instruments developed in the policy (see Table 7).

**Table 7:** Quantitative standard of the policy tool.

Policy tools	Detailed classification	Meaning	Scoring and quantification criteria
Authoritative tools G1	Laws and Regulations	Compliance with the relevant laws and regulations. The government formulated a series of laws, regulations, and management regimes to progressively promote the openness of government data, maintain national security, protect personal privacy, and improve data utilization.	5 Measures are formulated for policy tools from various angles, and the content is very detailed. 4 Measures are formulated from various perspectives of policy tools, and the content is more detailed. 3 Active support is provided from some aspects of the policy tool, and the content is relatively detailed, or active support is provided from a particular aspect, and the content is very detailed. 2 It is only from the attitude of certain aspects or aspects of policy tools that the relevant measures are actively used, but with no explicit content formulated. 1 The use of relevant measures is only specified from certain aspects such as attitude or the policy tool, but with no detailed explanation provided.
	Assignment of rights and responsibilities	The organizational relationship between government departments and their responsibilities and functions are clarified.	
	Supervisory review	The government conducts oversight and review of open government data.	
Incentive tools G2	Infrastructure Construction	The infrastructure is provided to support the openness of government data, including the construction of government cloud platforms, government networks, big data centers, shared platforms, and open platforms.	
	Encouragement and guidance	The government encourages the active use of government data through policy or institutional means by government departments, enterprises, the public, etc.	
	Capital investment	The government offers financial support by granting special funds, indirect investment, capital subsidies, R&D funds, and construction funds.	
Capacity building tools G3	System Planning	The overall planning, development, and implementation of policies from a holistic perspective.	
	Talent Cultivation	The training and management of personnel are strengthened in terms of government information disclosure and data openness.	
	Safety and Security	After the disclosure of information, the security of the platform and data is ensured.	
System change tools G4	Organizational Structure	Mechanism and system reform and adjustment are carried out for the organizational system construction of government data openness.	
	Authority allocation	The adjustment and change of power.	

#### 4.3.2 Quantitative criteria for policy targets

The policy subjects are assigned a value from 1 to 5 depending on their attitudes, measures, and effects on the target. Higher scores indicate stronger attitudes, more explicit goals, and more apparent effects of the policy subject (see Table 8).

**Table 8:** Policy target quantitative standard.

Policy targets	Detailed classification	Scoring and quantification criteria
Promoting data openness M1	Based on the perspective of the content included in the government’s data openness policy, we explain the promotion of data openness from both internal and external aspects: One is to vigorously promote data sharing among different government departments; The other is to progressively promote the openness of external public data resources possessed by the government.	5 There is a separate list of one or more policy objectives, and task details, a particular program is developed, and the openness of government data plays a significantly positive role. 4 One or more policy targets and tasks are listed separately, and a more specific program is developed, which significantly affects the openness of government data. 3 There is no separate list of one or more policy targets or task provisions, despite a clearly expressed objective in the policy. 2 There is no separate list of one or more policy objectives, task details, and objectives are expressed in the policy but without specific descriptions. Alternatively, there is a separate list of policy targets without specific programs developed. 1 The policy does not specify the policy targets for the research in this paper except for highlighting the need to accelerate the openness of government data in a certain way.
Deepening livelihood-related services M2	Promote the universality and accessibility of information services for better livelihood, offer data services for the public, and promote the value creation, public welfare, and innovative development and utilization of open data by society, contribute to the construction of inclusive livelihood, accelerate the development of new business model of livelihood services. Firstly, a city-wide unified open government data platform is established, an open data directory and standard specifications are developed, and different types of government data are opened to safeguard security and personal privacy. Furthermore, government data sets are gradually opened up in various fields such as transportation, commercial services, taxation, social credit, social security, health care, education, meteorology, environment, safety supervision, statistics, etc., for the provision of socially-oriented government data services.	
Building smart cities M3	Based on two aspects of cooperation and deep integration to start, Firstly, Expand the scope of openness, strengthen the cooperation with the smart city operation and management center, explore the socialized operation of data opening. Secondly, based on a unified government cloud platform, we strengthen the sharing and interoperability of city data and create smart government services. Thirdly, Accelerate the in-depth integration of artificial intelligence with public services and urban management, focus effort on promoting the construction of smart cities such as intelligent government affairs and innovative city management, and promote the modernization of social governance.	
Innovating social governance M4	Firstly, we should take scientific and technological innovation as a grip, promote social governance model innovation with big data and other information technology, and promote open sharing of data resource system; Secondly, shape a digital society, introduce Internet technologies, new applications, and new models, and improve the ability of scientific decision-making and social governance for the government. Finally, With the deepening of application and highlighting effectiveness as the priority of development foundation on, the open sharing of government data is accelerated, the application of big data is strengthened, and social governance models is improved.	

#### 4.3.3 Quantitative criteria for policy effectiveness

According to the type of policy and the level of the policy issuing agency, the effectiveness of the government data openness policy is assigned a value of 5, 4, 3, 2, or 1, respectively, from strong to weak, with a higher scores rep indicating greater effectiveness (see Table 9).

**Table 9:** Policy effectiveness quantitative criteria.

Score	Quantification criteria
5	Local regulations promulgated by the National People’s Congress and its Standing Committee
4	Regulations promulgated by the government
3	Temporary regulations, plans, decisions, opinions, measures, detailed rules, plans, and standards promulgated by the government
2	Opinions of committees, bureaus, and offices under the jurisdiction of the government
1	notices, announcements, etc.

### 4.4 Policy scoring

#### 4.4.1 Text encoding

(1) Category code

For the 85 policy documents collected for this paper, the policy instruments and policy targets are first categorized and tagged for coding (see Tables 10 and 11), while the contents of the collection of policy texts are coded and analyzed in line with the corresponding set rules applied to the subsequent work of this paper.

**Table 10:** Policy tool type code.

Number	Policy tools	Category number
1	Authoritative tools	G1
2	Incentive tools	G2
3	Capacity building tools	G3
4	System change tools	G4

**Table 11:** Policy target type code.

Number	Policy targets	Category number
1	Promoting data opening	M1
2	Deepening people’s livelihood services	M2
3	Building smart cities	M3
4	Innovating social governance	M4

(2) Content coding

The collected policy texts are analyzed through content coding. For each policy, the smallest paragraph is treated as a basic unit of analysis, whose number is sorted according to the specific content of the policy, i.e., a chapter number—sequence number. Then, the corresponding policy tool is matched with the serial number of the policy objective according to the content of that unit of analysis. The policy coding is performed in the form of “policy number—unit No. (Chapter No.—Serial No.)—Policy Tool/Policy Goal No.”. For example, 1-1-2-M1 indicates that the unit of policy content analysis as represented in a particular policy is derived from the second rule in the first significant category with a policy number of 1 and that the relevant content is the objective M1 in the category of policy objective.

#### 4.4.2 Quantitative scoring

**Fig 5:**
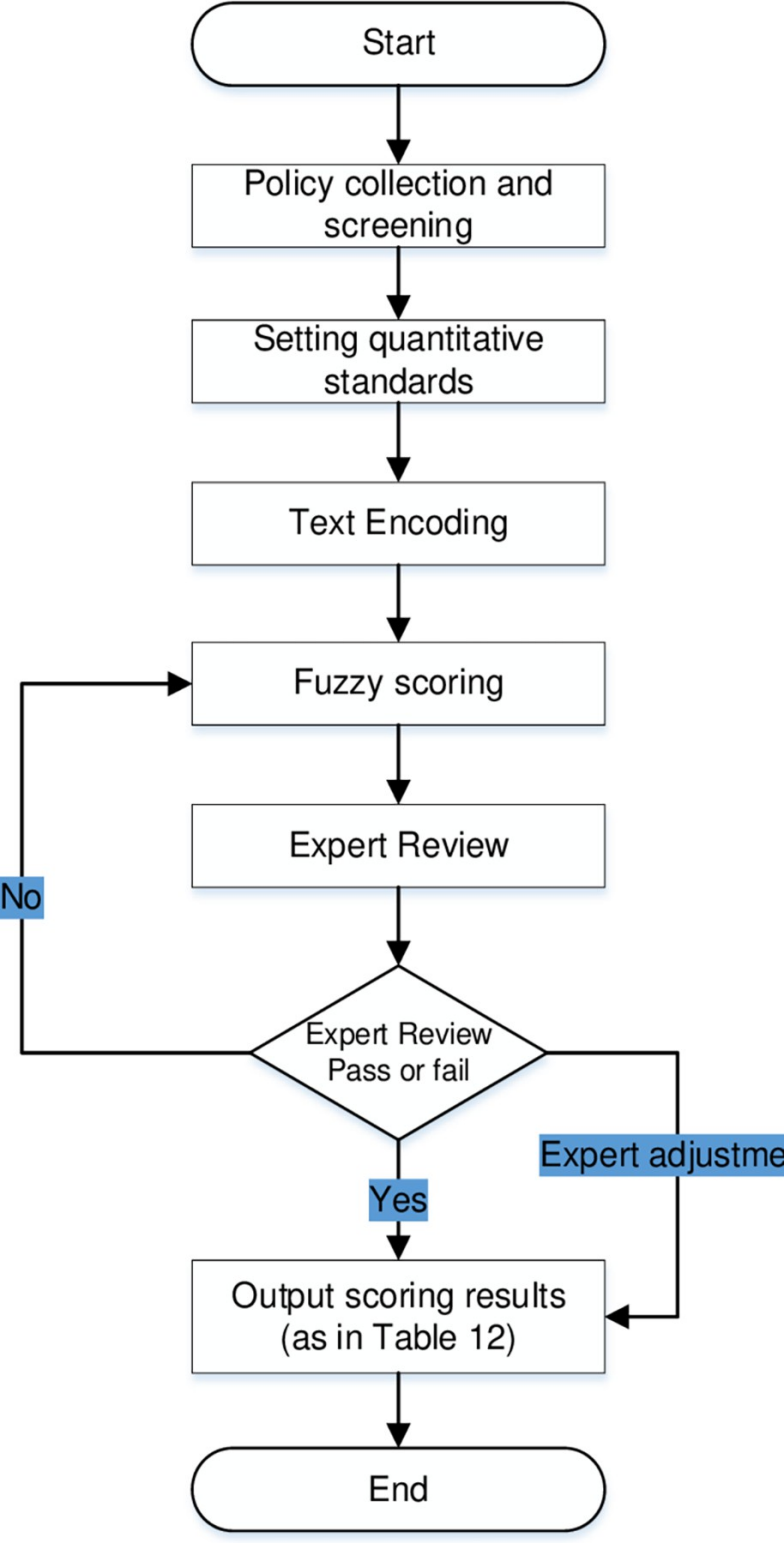
Quantitative scoring process for policies.

By applying the policy measurement criteria, fuzzy comprehensive evaluation and expert review are conducted in this paper for scoring. In line with the scoring criteria, each policy is scored in terms of policy strength, policy tools used, and policy targets according to the arithmetic mean of the first-level indicator scores. On this basis, a flow chart is drawn for the scoring based on the coding rules (see Fig 5).

Firstly, scoring is completed in line with the quantitative criteria, which is based on policy coding. To ensure the accuracy and validity of policy coding and quantification, a team of three is assigned for decision-making. This team is divided into three groups. In case of no consensus reached after group discussion, it would be referred to the other two groups for further discussion (see Fig 4).

Secondly, the final quantitative results of the policy instruments are calculated for each policy. To begin with, the scores of the policy instruments are calculated as a weighted average, which is based on the aforementioned scoring of the first-level indicators. If there is a policy with multiple scores for one level-1 indicator, the arithmetic average of these scores is taken as the final score of the corresponding instrument, thus obtaining the final scores of the four policy instruments directly. The final scores of each policy in terms of authoritative, incentive, capacity building, and systemic change tools are the arithmetic averages of the final scores obtained for the four level-1 indicators.

Finally, the final quantitative results of the policy targets are calculated for each policy. With no subgoals established for the policy objectives, they are grouped into broad categories according to the four primary objectives and scored. The final score of each policy objective is the average of their four respective categories in terms of promoting the open sharing of government data, deepening livelihood-related services, promoting the construction of intelligent cities, and innovating social governance (Table 12).

**Table 12:** Policy sample coding and quantitative scoring.

Number	Policy name	Unit number	Policy content	Content code	Policy effectiveness	Policy tools	Policy targets
1	Xi’an City to promote the coordinated development of an e-government implementation plan	1–2	(2) The main goal. By 2020, the completion of the e-government network will be in line with national requirements, and the regional e-government network to achieve security docking. Network information security guarantee capability is significantly enhanced, information sharing, business collaboration, data opening, and data depth utilization level is significantly improved, the service government decision-making and management of information technology capabilities significantly improved, the government public services online operation is entirely popularized, e-government in the city’s governance system and the role of modernization of governance capacity to further appear.	1-1-2-M1	3		4
		2–1	(1) Speed up the construction of the e-government network.	1-2-1-G2	3	3	
		2–2	(2) Strengthen the top-level design, and coordinate the coordinated development of e-government.	1-2-2-G1	3	2	

### 4.5 Synergistic measurement model

#### 4.5.1 Policy effectiveness measurement model

Policy effectiveness size indicates the level of institutional hierarchy for the issuing policy subject. It is divided into total policy effectiveness and average policy effectiveness, both of which are the indicators used to reflect the total policy effect and average policy effect in a specific period under the combined effect of policy strength, policy instruments, and policy objectives, respectively. The evaluation of policy targets and measures is to quantify the clarity and strength of the policy and to measure the synergy of effectiveness against the three indicators, which addresses the deficiency of using individual indicators to reflect the effectiveness of the policy [34]. Therefore, Eq (1) is used to calculate the total effectiveness of government data openness policies for each year, and Eq (2) is used to calculate the average effectiveness of government data openness policies for each year.

$${YTPE}_{i}=\sum_{j=1}^{N}{pe}_{i,j}\times {pt}_{i,j}\times {pg}_{i,j}\;i=[2015,2021]$$ (1)

$${YPE}_{i}=\frac{\sum_{j=1}^{N}{pe}_{i,j}\times {pt}_{i,j}\times {pg}_{i,j}}{N}\;i=[2015,2021]$$ (2)

where i is the year, i = [2015, 2021], N is the number of policies in year i. and are the total and average effectiveness of government data openness policies in the year I, and are the policy strength score, total policy instrument score, and total policy goal score of policy j in the year i, respectively.

#### 4.5.2 Policy tool synergy measurement model

Policy tool synergy is a measure of the use of multiple policy tools in a single policy, and generally, the stronger the policy and the more explicit the tools used, the higher the synergy. This section mainly draws on Jisheng Peng’s science and technology innovation policy [34] to construct a policy tool synergy measurement model for government data openness. Therefore, Eq (3) calculates the synergy between two government data openness policy tools for each year.

$${PTC}_{i}=\sum_{j=1}^{N}{pe}_{i,j}\times {pt}_{i,j,k}\times {pt}_{i,j,l}\;i=[2015,2021]$$ (3)

where i represents the year; i = [2015, 2021]; N indicates the number of policies in a year I; denotes the synergy of government data openness policy tools in year i; is referred to as the policy strength score of the jth policy in a year i; and represents the score of the kth and lth policy tools in the jth policy in a year i, where k and l (k ≠ l) denote two tools (= 6) selected from four types of policy tools including authority, incentive, capacity building, and system change to analyze the synergy of government data openness policy tools.

#### 4.5.3 Policy target synergy measurement model

The synergy of policy targets is to reflect the planning of multiple objectives under one policy. In general, the stronger the policy and the more precise the objectives published, the more significant the synergy. Therefore, Eq (4) is used to calculate the degree of synergy for each annual goal of government data openness policy.

$${PGC}_{i}=\sum_{j=1}^{N}{pe}_{i,j}\times {pg}_{i,j,m}\times {pg}_{i,j,n}\;i=[2015,2021]$$ (4)

where i is the year, i = [2015, 2021], N is the number of policies in a year i, denotes the synergy of government data openness policy targets in year i, denotes the policy strength score of policy j, and is the score of policy targets m and n in policy j in year i. where, m and n(m ≠ n) denote two objectives (= 6) selected from four policy objectives, such as promoting data openness, deepening livelihood services, building smart cities, and innovating social governance, to analyze the two-two synergy between government data openness policy objectives.

## 5 Synergistic analysis of data opening policies in regional central cities

### 5.1 Volume and effectiveness evolution

With the orderly promotion of China’s big data strategy, the government’s attention to data openness is also increasing. China implements the management level of “one central government, one province, and one city.” Under the guidance of the central government, the number of data-opening policies issued by cities is also increasing, and the total effectiveness of procedures is also increasing. The number and points of policies in regional central cities show a significant fluctuation in different years.

**Fig 6:**
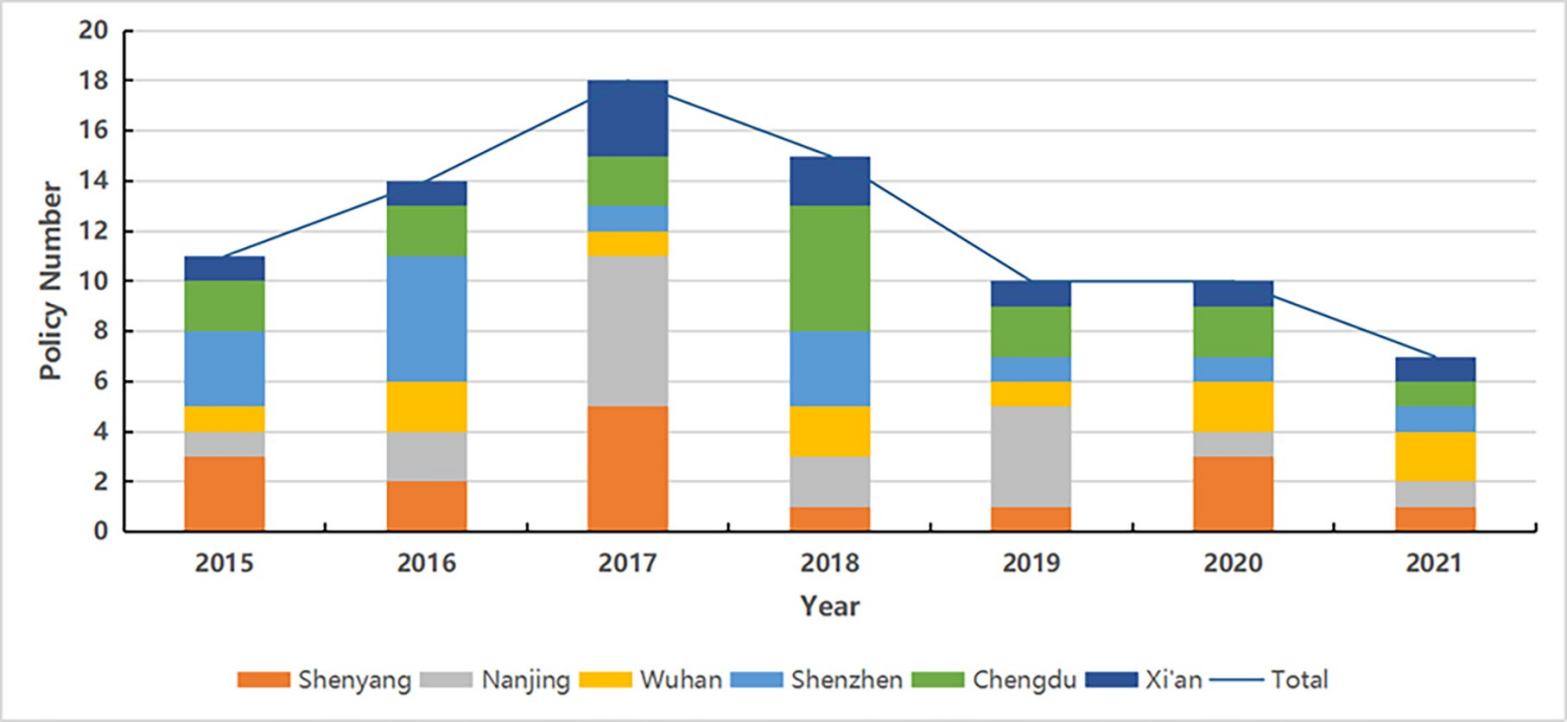
Analysis of policy quantity evolution.

(1) Analysis of policy quantity evolution (see Fig 6)

Since 2015, the number of policies in regional central cities peaked in 2017, which is due to the central government’s promulgation of some essential strategic planning documents and policy documents after 2015, such as the action platform for promoting the development of big data in 2015 and the implementation plan for the integration and sharing management of government information system in 2017. Overall, due to the decline of central guidance documents in 2018, the response of municipal governments decreased, resulting in a decline in the number of policies.

(2) Analysis of the evolution of policy effectiveness

**Fig 7:**
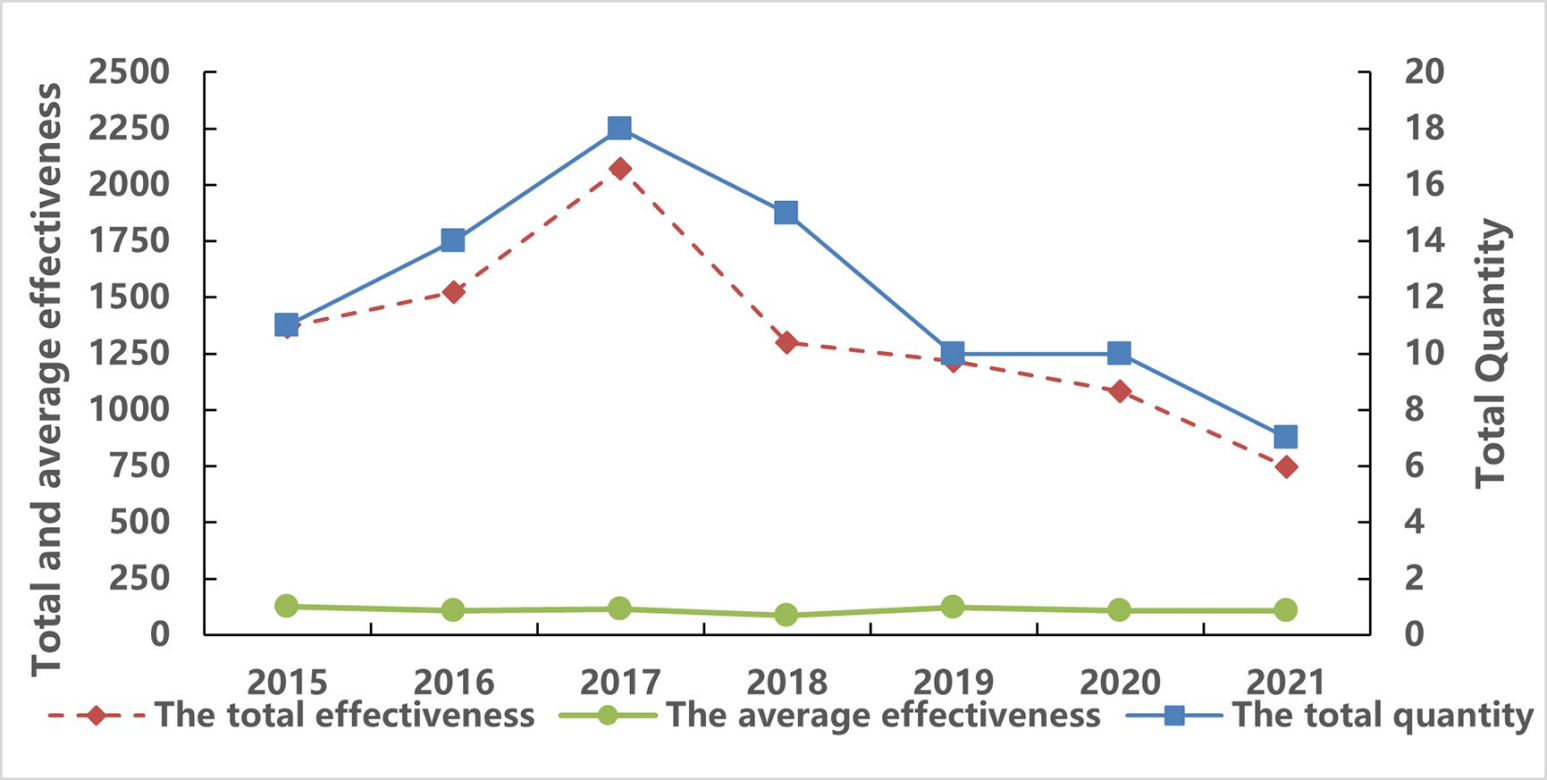
Evolution of data openness policy total effectiveness, average effectiveness, and policy quantity.

Through the analysis of the total effectiveness of the data opening policy of regional central cities (see Fig 7), it is found that the total effectiveness and quantity of the policy show a positive correlation trend between 2015 and 2021; However, the average effectiveness of the policy does not have an upward curve and tends to be stable. It can be seen that the effect of policy release has not reached the expected effect, and the effectiveness of policy release is weak.

### 5.2 Analysis of the synergistic evolution of policy tools

**Fig 8:**
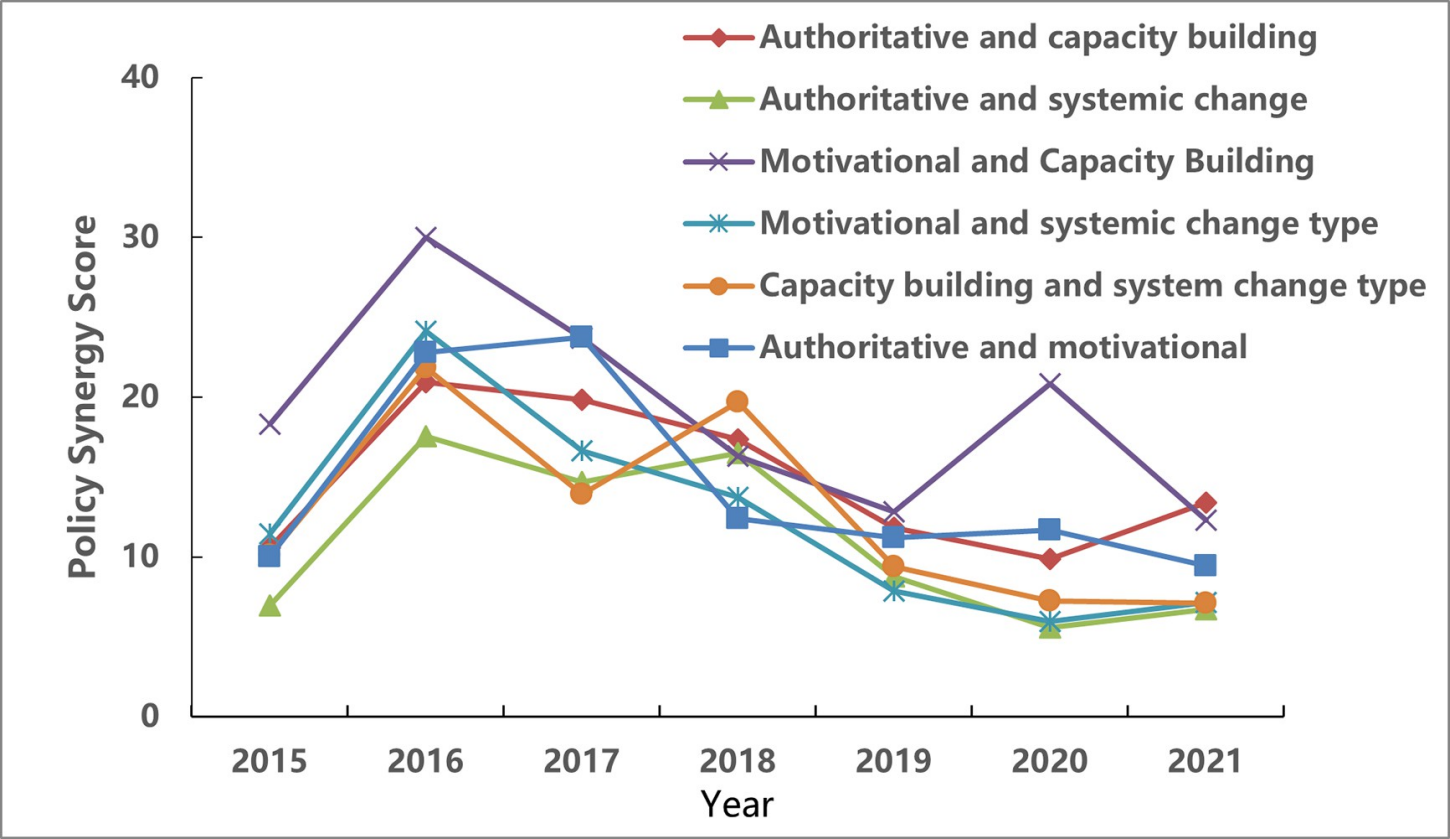
Analysis of the evolution of synergy among authoritative, incentive, system-changing, and capacity-building policy tools.

The Policy Instrument Collaborative Measurement Model constructed above calculates the synergy degree of policy tools to reveal the collective evolution of data-opening policy tools in regional central cities. Use Formula (3) to calculate the synergy between two policy tools in regional central cities (see Fig 8).

It can be seen from the figure that the synergy of the six policy tools shows periodic fluctuations and the synergy degree reached its peak in 2016. This is due to the promulgation of many government data-opening policies in the above years, especially the everyday use of multiple policy tools, which has driven municipal government policies. Horizontally, the collective evolution between policy tools can be roughly divided into two periods the growth period (2015–2017) and the stable period (2018–2021). Specifically, from 2015 to 2017, the synergy score of Government Data Opening Policy tools generally increased first and then decreased, and the synergy score of incentive and capacity-building tools reached the maximum in 2016; From 2018 to 2021, the synergy score of tools generally showed a downward trend, but the synergy score of incentive and capacity-building tools remained at a high level.

### 5.3 Analysis of the synergistic evolution of policy targets

**Fig 9:**
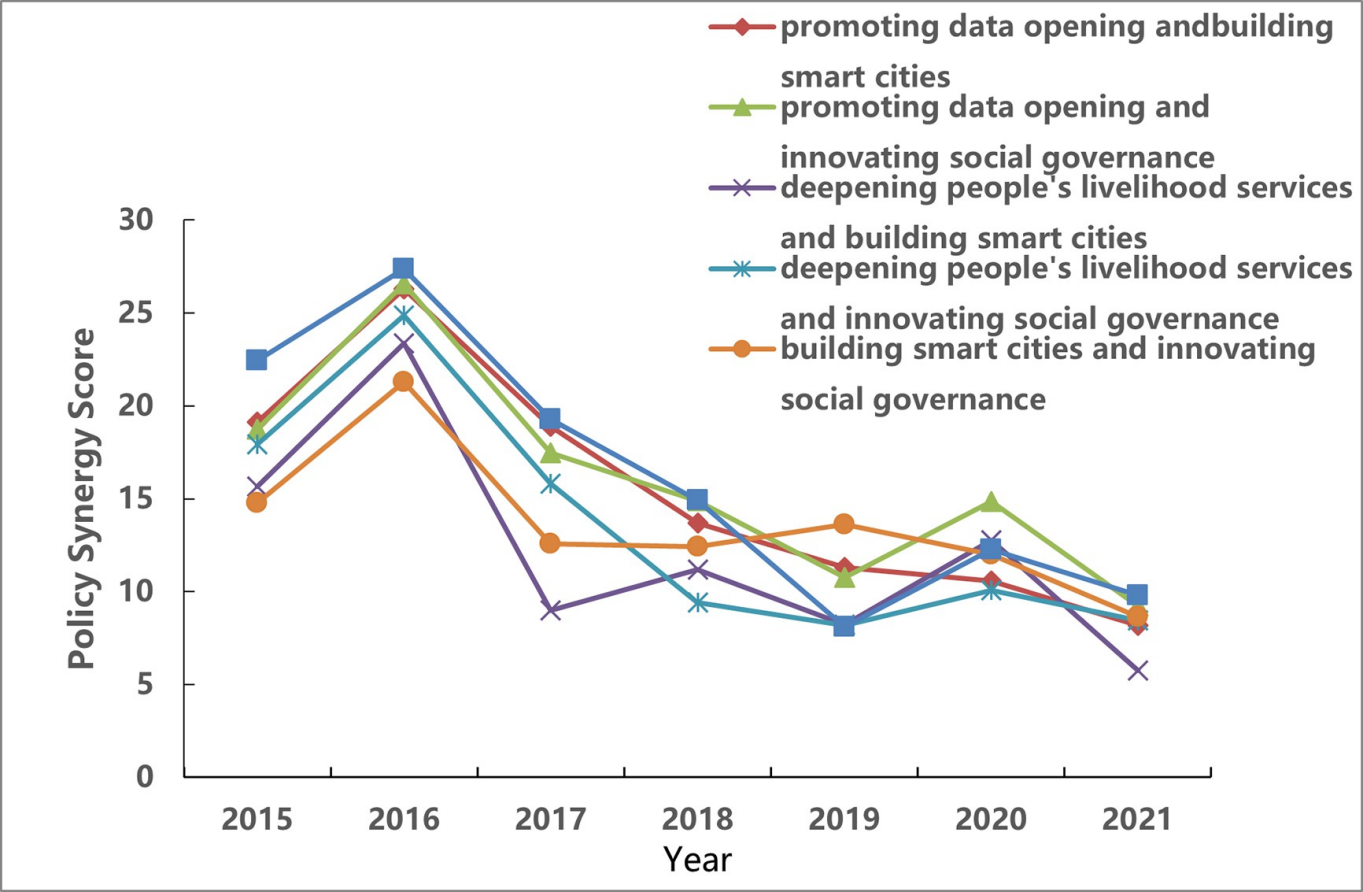
Analysis of the evolution of synergy among policy targets of promoting data opening, deepening people’s livelihood services, building smart cities, and innovating social governance.

The Policy Goal Synergy Measurement Model constructed above calculates the synergy degree of policy targets to reveal the collective evolution of data-opening policy targets in regional central cities. Use Formula (4) to calculate the degree of synergy between two policy targets of regional central cities (see Fig 9).

As shown in the figure, the synergy of the four policy targets is shown as the change from increase to decrease to steady. In particular, promoting government data openness is above the other three goals, indicating that promoting data openness drives the development of other goals. Horizontally, the coordinated evolution between policy targets can be divided into the growth period (2015–2017) and the stability period (2018–2021). Specifically, from 2015 to 2017, the synergy score of the government’s data opening policy targets generally increased first and then decreased, and the synergy score of promoting data opening and deepening people’s livelihood service objectives reached the maximum in 2016; From 2018 to 2021, the synergy score of policy targets fluctuates within a specific range and tends to be stable. The synergy score promoting data openness and innovative social governance will reach the maximum in 2020.

## 6 Conclusions and suggestions

### 6.1 Conclusions

(1) Firstly, the changing trend of the number of data opening policies in my country’s regional central cities and the total policy effectiveness is roughly the same, showing periodic up and down fluctuations, and the total policy effectiveness and the number of policies are positively correlated, indicating that the number of policies promulgated by the government at the municipal level government plays a positive role in promoting the level of data openness to some extent, the average effectiveness of policies has not shown the expected upward trend, and is almost entirely stable, which proves that the policies enacted by the government at the municipal level are not well targeted.

(2) Secondly, the synergy between policy tools fluctuates wildly, indicating that governments at the municipal level are experimenting with different approaches to promote open government data. In addition, the synergy between incentive tools and capacity-building tools is always higher than that between other tools, which also proves that the government focuses on capacity-building and incentive measures simultaneously and is in a dominant position. Overall, the municipal government can use incentive tools and capacity-building tools to formulate data-opening policies, but the coordination of authoritative and systematic change policy tools needs to be strengthened.

(3) Thirdly, the coordination of policy targets shows a changing trend of first rising, then falling, and then stable. With the promotion of various work, the synergy between the promotion of data opening objectives and the other three objectives is higher than that among other objectives, which proves that the promotion of data opening objectives has a specific promoting effect on other target areas and also shows that the data opening objectives are also the basis of other objectives. However, overall, the synergy between the objectives of deepening livelihood services and building smart cities in the follow-up work of the governments at the municipal level needs to be further strengthened.

### 6.2 Suggestions

According to the analysis conclusion of the data opening policy text of regional central cities, and referring to the ranking of China’s open forest benchmark cities in China’s local government data opening report (the first half of 2021), learn from the practice of Shanghai and Guiyang, which rank first and fourth, to provide corresponding suggestions and Countermeasures for strengthening the development of data opening of municipal governments in China.

(1) Moderately improve the effectiveness of data opening strategies and enhance the implementation of policies. China’s municipal government should pay more attention to the opening of government data, strengthen the intensity control of relevant departments, reasonably allocate personnel and improve the efficiency of policy implementation. For example, Guiyang City the first place in China to legislate big data and the first prefecture-level city in China to launch the government data opening platform. It issued the Guiyang action platform for big data in 2016 and formulated policies such as the regulations on the sharing and opening of government data and the regulations on the security management of big data in 2021 to standardize and stipulate issues such as data collection, opening and sharing, and data security. By implementing the Interim Measures of Shanghai Municipality on public data openness, Shanghai has formulated special laws to provide a complete institutional guarantee for government data disclosure and public data sharing to improve data openness effectively.

(2) Strengthen the coordination between authority and systematic change policy tools to balance the differences between the use of tools. Strengthen the coordination of authoritative and systematic policy tools, strengthen the mandatory tools of municipal governments, and pay attention to the adaptation of internal government reform to data opening. First, we should actively use relevant channels and measures to improve them for authoritative tools. At the same time, we should improve the establishment of laws and regulations, supervision and review, clarify the distribution of rights and responsibilities, and effectively implement the data opening policy; Secondly, for system change tools, strengthen the collaborative use of organizational structure and power and responsibility distribution; In short, only by comprehensively utilizing the joint driving force of various policy tools, releasing data dividends and realizing data value-added, can we better improve the level of government data openness. For example, Guiyang has made extraordinary efforts to clarify the distribution of corresponding rights and responsibilities by formulating a plan to promote the headquarters’ establishment for the sharing and opening of government data. Shanghai has formulated policies such as the Shanghai data regulations (Draft) to provide a fundamental guarantee for the digital development of the government, set up regulations to standardize further and promote the sharing of government information resources and the opening of public data resources and strengthen the management of government information resources.

(3) Further clarify the objectives of the government’s data opening policy and strengthen the tightness of the objectives. All municipal governments should pay attention to the infrastructure and related conditions of their respective cities, find the region’s development goals according to the local conditions, and carry out the sustainable development and adjustment of the goals. In addition, in the follow-up policy objective planning process, the government should focus on building a smart city and deepening the coordinated application of people’s livelihood service objectives. On the one hand, building an intelligent city and deepening people’s livelihood services should be set according to specific objectives and the specific conditions of each city to meet the needs of public social data; On the other hand, responding to the municipal data opening objectives and essential documents issued by the central government, make specific objectives according to the market demand analysis, and speed up the market-oriented construction of data elements. For example, the “ten-year plan” will be released in January 2022.

In the context of the fourth five-year plan for digital economy development, Guiyang is expected to implement the “five actions” in 2022, cultivate new driving forces for extensive data development, deepen people’s livelihood services, accelerate urban digital construction, and improve the strength of the digital economy. In October 2021, Shanghai released the 14th five-year plan for comprehensively promoting urban digital transformation, which proposed to achieve the urban digital governance of “multi governance” of government, market, and society by 2025, preliminarily realize the overall transformation of production and life, realize the interconnection of cross-industry, cross-level, and cross-system data, promote the integration and application of public data and social data, and strive to realize the digital transformation of the city.

## 7 Deficiencies and prospects

By constructing a three-dimensional analysis framework of “policy tools-policy objectives-policy intensity” of municipal governments, this paper quantifies the text of government data opening policy in regional central cities. It conducts a collaborative evolution analysis and discussion, which enriches the coordination of data opening policies of municipal governments in the country—related research in measurement. However, there are still some deficiencies in this study: First, the policy texts collected are from regional central cities in the country, and the sample of policy texts is limited, so we can try to collect more policy texts from municipal governments in the future; second, the data opening policy has not been studied yet. In the future, we can explore the influencing factors and implementation effects of policy coordination on the data opening of municipal governments and conduct more profound research. In the future, the data opening policy text of the municipal government can be collaboratively entered into in-depth research, and the above shortcomings are also the focus of follow-up research.

## Supporting information

S1 Annex (XLSX)

S2 Annex (XLSX)

S3 Annex (XLSX)
